# Proteomic tools to decipher microbial community structure and functioning

**DOI:** 10.1007/s11356-014-3898-0

**Published:** 2014-12-05

**Authors:** Florence Arsène-Ploetze, Philippe N. Bertin, Christine Carapito

**Affiliations:** 1 Génétique moléculaire, Génomique et Microbiologie, Université de Strasbourg, UMR7156 CNRS, Strasbourg, France; 2 Laboratoire de Spectrométrie de Masse Bio-Organique, Institut Pluridisciplinaire Hubert Curien, UMR7178 CNRS, Université de Strasbourg, Strasbourg, France

**Keywords:** Community proteomics, Metaproteomics, Mass spectrometry, Targeted proteomics, LC-SRM, Quantitative proteomics

## Abstract

Recent advances in microbial ecology allow studying microorganisms in their environment, without laboratory cultivation, in order to get access to the large uncultivable microbial community. With this aim, environmental proteomics has emerged as an appropriate complementary approach to metagenomics providing information on key players that carry out main metabolic functions and addressing the adaptation capacities of living organisms in situ. In this review, a wide range of proteomic approaches applied to investigate the structure and functioning of microbial communities as well as recent examples of such studies are presented.

## Introduction

Microbial communities are complex biological assemblies, whose study has been difficult for a long time because a large fraction of the species is unknown (Bertin et al. 2008). Indeed, in any given environment only a small fraction of organisms present can actually be cultivated. Nowadays, these communities can be explored as a whole by using environmental genomics. Genomics aims to study the biology of microorganisms by analyzing the genetic information they contain. The concept of metagenomics, i.e., sequencing of the genomic DNA content of a community, has emerged over the past 15 years and provides a detailed inventory of genes from a community and thus of the potential capacities of microorganisms (Bertin et al. 2008). A significant reduction in sequencing costs combined to new broadband technologies has led to an explosion of such genomic programs. These approaches have resulted in an inventory of the genomic content of several communities but have also highlighted the high diversity found in nature (Schleper et al. 2005; Bertin et al. 2008; Lasken 2012; Parkhill 2013; McCann et al. 2014; Tseng and Tang 2014). To go further in the understanding of community functioning, these approaches are now often linked to functional genomic approaches allowing the characterization of genes expressed in an organism under specific conditions. Two approaches are commonly used in functional genomics of communities: metatranscriptomics to study messenger RNAs and metaproteomics or whole community proteomics to characterize proteins.

The metaproteomics approach has some advantages when compared to metatranscriptomics. Indeed, as proteins are more stable than RNAs (especially those originating from prokaryotes), the metaproteome content is supposed to be less affected by extraction procedures, and probably gives a better insight into the biological functions expressed in situ. Moreover, proteins are crucial effectors of the biological response of living organisms. The amount of these effectors in organisms varies at different levels and a modulation of their activity depends on a change of the corresponding genes expression, post-translational modifications, or proteolysis/protein turnover. Therefore, there has been a growing interest in studying expression of proteins of various microorganisms in their habitats and, in the field of microbial ecology, metaproteomics (Wilmes and Bond 2004) or community proteomics (Ram et al. 2005; Lacerda and Reardon 2009; Keller and Hettich 2009) has emerged to characterize in a global way the protein content of microbial communities (Hettich et al. 2012, 2013). Metaproteomics is used to obtain protein catalogues giving important information on the community activity or its structure, but also to compare protein contents in two different ecosystems by using quantitative metaproteomics or to complete or correct metagenomic data (community proteogenomics). In this review, global proteomic approaches used to decipher the physiology of a microorganism or the functioning of microbial communities will be presented with a particular focus on recent advances in global and targeted quantitative proteomics approaches.

### Proteomic analysis workflow

**Fig. 1:**
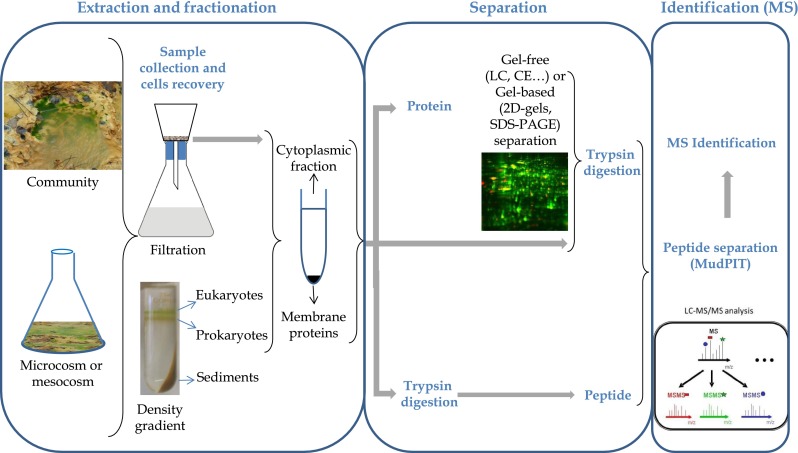
Proteomic workflow to study the structure, functioning, and interactions in complex communities

The proteomic approach was first defined as a functional genomics approach allowing studying the protein expression pattern of one organism, i.e., to obtain a protein map of all proteins expressed by one organism grown in one particular condition. Such an approach was then developed to compare proteins expressed by an organism incubated in two different conditions. This approach, called differential proteomics, requires quantifying protein amounts in each condition. Proteomics was thus complementary to genome sequencing, giving information on the non-model microorganism activities (Muller et al. 2007; Weiss et al. 2009). Finally, such an approach is now used at the community level (metaproteomics). The success of (meta)proteomic workflows depends essentially on three crucial steps: the efficiency of proteins extraction, the methods used to separate or fractionate proteins in a complex mixture, and the unambiguous identification of peptides/proteins from tandem mass spectrometry (MS/MS) data (Fig. 1). In addition, robust quantification methods are required in order to compare expression patterns in different conditions.

#### Protein extraction

To study the proteins expressed by microorganisms, a sufficient quantity of cells has to be collected in order to extract their protein content. Indeed, since there is no “PCR-analog” for proteins, enough material has to be extracted to allow the detection and identification of proteins without any “amplification” step. At least 20 to 50 μg of proteins are typically required to conduct a proteomics experiment. Working with lower amounts of material is delicate and often leads to very poor peptide recovery for further liquid chromatography-MS (LC-MS) analysis after enzymatic digestion and desalting. On most sensitive nanoLC-MS/MS systems, high attomole to low femtomole levels of peptides can be detected, lower limits obviously depending on the peptide mixture complexity and dynamic range. To reach a reasonable amount of starting protein material, the amount of samples to be treated directly depends on the characteristics of the studied site. As an example, in the sediments of the Carnoules AMD, 50 ml of sediments had to be treated to obtain around 10 μg of proteins (Arsène-Ploetze, unpublished data). Therefore, the cell collection step is crucial in metaproteomics and a low biomass collection often makes this approach unsuccessful. In the case of marine or freshwater context, cell collection is often performed by filtration (Fig. 1). The filtration often needs to be done on hundreds of liters of water to recover enough cells. When the community is within soil or sediments or associated to plants or animals, a preliminary step may be required in order to take off cells from biotic or abiotic surface or from surrounding particles. This step is particularly important in the case of microorganisms recovered from soil or sediments, which naturally contain interfering substances such as humic acids, inevitably extracted together with proteins (Bastida et al. 2009). Moreover, when the community is more complex, for example when it is composed of eukaryotes and prokaryotes, it may be necessary to split the different types of cells and to study only a fraction of the community (Fig. 1), or to preserve the cells for further analysis in laboratory. For example, the main oceanic microbial populations, namely *Synechococcus* cells, have been studied using proteomics after cell separation using microwave fixation and flow cytometry sorting (Mary et al. 2010). In several studies, it has been possible to separate microorganisms from sediments, but also bacteria from the eukaryotic population using a Nycodenz gradient (Fig. 1) and study the two populations separately (Bertin et al. 2011; Halter et al. 2012). Recently, successful protocols have been developed to extract proteins directly from soil without separating cells and particles (Chourey et al. 2010; Keiblinger et al. 2012). Unbiased protein extraction requires optimization of lysis conditions (Fig. 1). Several protocols are usually tested, such as those described in (Cañas et al. 2007). Physical lysis methods are the most commonly used in the case of microorganisms, such as the use of glass beads grinding, sonication, alternating cycles of freezing and thawing, or high- and low-pressure cycles. The combination of these methods with enzymatic lysis or the use of detergents can improve the efficiency of cell lysis (Cañas et al. 2007).

#### Protein separation

Proteins can be separated prior to identification in order to fractionate very complex protein mixtures and analyze each protein fraction separately. As the complexity of environmental samples is very high with proteins present at widely spread concentration ranges, this strategy is often chosen for metaproteomic studies. Protein separation can be achieved by different methods among which the most commonly applied are electrophoresis on acrylamide gel (1D-polyacrylamide gel electrophoresis, SDS-PAGE, or 2D-gel-electrophoresis, 2D-GE), capillary electrophoresis (CE), or liquid chromatography (LC; Fig. 1). Historically, 2D-GE separation was most successfully applied in proteomics studies (Rabilloud et al. 2010). Proteins are separated in a first step according to their pI and in a second step as a function of their molecular weight. After separation, proteins are usually visualized with an organic dye (Coomassie blue), by reduction of a metal salt (silver nitrate) or a fluorescent labeling (Sypro, DeepPurple, …). However, the 2D-GE approach has several limitations including the dynamic range of spots visible on the gels and the loss of membrane proteins due to their low solubility in 2D-GE solvents. For the preparation of membrane proteins requiring specific detergents, one-dimensional SDS-PAGE is often preferred (Laemmli 1970). Besides electrophoresis, gel-free strategies are now also commonly used to separate proteins such as multi-dimensional liquid or affinity chromatographies (Gundry et al. 2009). Many types and combinations of chromatographies have been explored in the field of proteomics to fractionate complex protein mixtures prior to identification. Each of these approaches has advantages and disadvantages and the choice of the best adapted separation method is highly dependent on the sample type to be analyzed. Early environmental proteomics researches used 2D-GE to separate proteins, but the achieved resolution was low (Wilmes and Bond 2004; Lacerda et al. 2007; Bruneel et al. 2011). Recent protocols were optimized to obtain better 2D-GE performances (Kuhn et al. 2011). SDS-PAGE or LC-MS/MS were also used (Benndorf et al. 2007; Wilmes et al. 2008a, b) allowing the authors to identify at least 10 times more proteins. In some cases, the high level of diversity of the communities and/or the wide dynamic range of abundances (both at the species as well as protein levels) make metaproteomics approaches rather difficult to apply. Therefore, for several years, metaproteomics studies were only successful when applied to communities with low levels of diversity. When a high level of diversity was observed, only the most abundant proteins could be identified. After protein separation and enzymatic digestion, the generated peptides are analyzed by MS and most commonly tandem MS (MS/MS) in order to precisely measure peptide masses and their associated fragments (in MS/MS mode).

#### Protein identification

The protein identification step of the general proteomics analysis workflow relies on the availability of high performance MS instrumentation generating sensitive and high resolution data but certainly also on sophisticated bioinformatic tools to extract most useful information from the generated data. The most widely used approach for protein identification is based on a prior enzymatic digestion of proteins into peptides and is thus called the “Bottom-up” strategy. “Shotgun” proteomics approaches can be used (Fig. 1) consisting in the straight enzymatic digestion of the complex protein mixtures without any prior fractionation; trypsin is the most commonly used enzyme which specifically cleaves the polypeptide chain after lysine and arginine residues. The peptides obtained are chromatographically separated by reverse phase liquid chromatography (LC) and analyzed by mass spectrometry (MS; Nesvizhskii 2010). Alternatively, a MudPIT (multidimensional protein identification technology) strategy can be applied, involving the use of multiple and various chromatographic separations of the peptides prior to their injection in the MS (Fränzel and Wolters 2011). The generated peptides are then analyzed by high-resolution mass spectrometers (MS), capable of ionizing and measuring peptide masses with a sub-ppm mass accuracy.

The general protein identification principle is based on the comparison of experimental masses to theoretical masses calculated from proteins present in protein sequence databases. Historically, the Peptide Mass Fingerprint (PMF) approach, based on tryptic peptides’ masses measurements and their comparison with peptides’ masses predicted from protein databases, was used to identify proteins. Though, this approach rapidly appeared to be not specific enough to unambiguously identify proteins because of the exponentially growing size of protein sequence databases. Nowadays, routine identification of proteins requires additional tandem MS (MS/MS) data, which allows accurately determining peptides’ masses and their associated fragments (MS/MS). Numerous algorithms have been developed over the last 10 years to extract most reliable identifications from large-scale and high-throughput LC-MS/MS data by matching experimental mass lists and MS/MS spectra with in silico calculated mass lists (Nesvizhskii 2010). Additionally, strategies to evaluate false discovery rates (FDR) using target-decoy databases have been implemented in order to constantly attest for the overall quality of protein identifications (Elias and Gygi 2007). With the constant increase of the MS/MS sequencing pace of mass spectrometers and of the size of protein sequence databases (as a direct consequence of next generation DNA sequencing and automatic annotation of genomes), the MS/MS data interpretation step becomes highly informatic-resources consuming. In order to circumvent this bottleneck that MS/MS data interpretation commonly represents, software using distributed computing resources such as cloud or grid solutions have been developed (Carapito et al. 2014).

When analyzing a bacterial community as a whole, the genomes of the studied organisms are often unknown. In this case, identifications can be performed by using de novo sequencing that consists in the interpretation of MS/MS spectra to derive an amino acid sequence tag from each individual MS/MS spectrum. The derived sequence tags are then subjected to MS-BLAST (http://dove.embl-heidelberg.de/Blast2/msblast.html) to search for sequence homologies with orthologous proteins present in the databases (Carapito et al. 2006). This identification process is more time consuming, less high throughput than classical protein database searches and often requires manual verification of sequence tags and MS/MS spectra quality. Additionally, potential post-translational modifications (e.g., glycosylation or phosphorylation) may also complicate de novo sequencing. However, latest generation MS instruments resolutions, accuracies, and fragmentation quality, together with enhanced algorithms allow increasing the throughput and successful identification rates and reducing the FDR of de novo sequencing approaches (Carapito et al. 2014). Once peptide sequences have been successfully identified by either one of the previous approaches, they have to be grouped to protein functions and in this step resides the big challenge of protein inference that is especially complex when rich bacterial communities, including many close organisms, are studied (Seifert et al. 2013).

In order to improve protein identification, metaproteomics and metagenomics are nowadays often combined (Seifert et al. 2013). It is crucial however that the metagenomics data are of good quality allowing a robust assembling of sequences in order to obtain a large quantity of gene sequences and almost complete sequences for each gene. The recent transition in DNA sequencing from Sanger to next generation sequencing (NGS) approaches may reduce the efficiency of metaproteomics identification rates, since the assembly of genomic sequences via NGS is limited due to the short reads obtained. In some cases, although genome information is available, the identification of proteins can be compromised due to errors in sequence databases (frameshift, false start codon prediction), which is often the consequence of the automatic annotations of genomes. To avoid these problems resulting from genome data that are not expertly annotated, identification strategies to interpret MS/MS data directly in the complete, unannotated genome sequence have been developed. These approaches are at the origin of the proteogenomics field, defined as the use of proteomics results to improve genome annotations (Delalande et al. 2005; Gallien et al. 2009; Armengaud et al. 2014). Such an approach may be combined with labeling methods such as specific labeling of protein N-termini, allowing the identification of peptides corresponding to protein starts. For instance, such an N-terminomics strategy has been used to check or correct errors of translation initiation codon prediction or to validate signal peptides for secreted proteins in the case of microorganisms belonging to poorly documented genera (Bertaccini et al. 2013).

#### Protein quantification

**Fig. 2:**
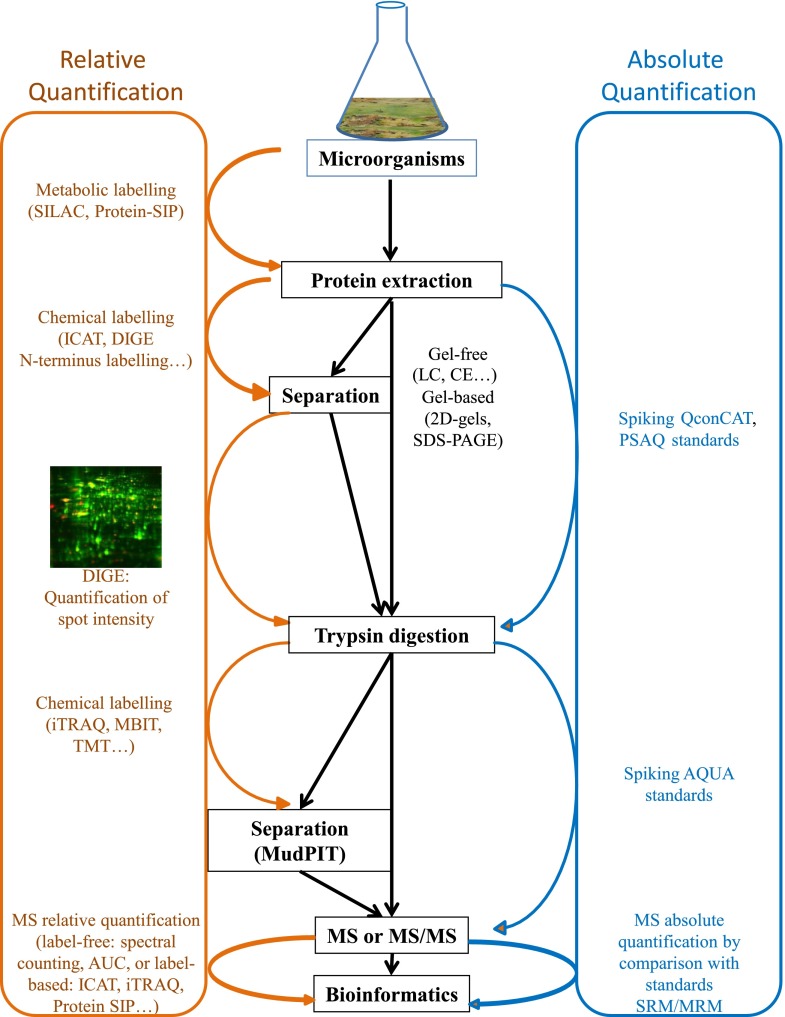
Protein quantification strategies. Quantitative methods can be either relative or absolute

Quantitative methods can be either relative or absolute (Fig. 2). Relative quantitative studies aim at determining the relative amounts of given proteins across different protein extracts, for instance across different growth conditions. A wide range of methods for relative quantification that differ in their accuracy, applicability, and sensitivity have been developed. Relative quantification methods do not allow intra-sample comparisons of abundances of peptide A to peptide B for example, as LC-MS response factors are highly variable from one peptide to another and depend on the peptides’ sequences. On the contrary, absolute quantification implies that concentrations of peptides/proteins are determined thanks to the comparison of signal intensities with highly purified/quantified isotopically labeled peptides/proteins, ideally spiked into the samples in known amounts. Absolute quantification methods are most precise and robust when applied with limited sample fractionation, thus preferentially using gel-free approaches as described below. The most widely used approach today for absolute quantification is targeted proteomics using selected or multiple reaction monitoring mass spectrometry (SRM or MRM) as described at the end of this review (Fig. 2).

##### Relative label-free quantification methods

Label-free quantitative approaches compare peptide spectral counts or peptide intensities between LC-MS/MS runs of samples (Nahnsen et al. 2013). The first approach, called spectral counting, relies on counting the number of MS/MS spectra acquired for a given protein. The second approach, called feature-based quantification, consists in calculating the relative intensities of extracted ion chromatograms (area under the curve, AUC) obtained using computational tools. These two approaches assume that the number of peptides identified for each protein, as well as their chromatographic intensity reflects the amount of protein present in each sample. Such approaches are useful when large numbers of samples are studied. However, they require high-quality and highly reproducible LC-MS. Because the samples are analyzed separately, this quantification may not be appropriate when small variations of protein amounts have to be observed between samples. Label-free methods have been used to quantify proteins from metaproteomic samples, using either signal intensities measured by MS for each peptide, spectral counts, or normalized spectral abundance factors (NSAF). For example, the amounts of proteins were compared using AUC in communities in aquifer during biostimulation (Callister et al. 2010) or by spectral counting on the same type of community (Wilkins et al. 2013). Such quantifications have also been performed to study the community found in oceans, in response to different nutrient concentrations (Morris et al. 2010), in AMD biofilm samples (Mueller et al. 2010), during litter decomposition (Schneider et al. 2012; Table 1). Recently, normalized intensities for each protein were calculated to quantify key markers in the field of human microbiome studies (Ferrer et al. 2013). Label-free quantification has also been used to estimate the efficiency of protein extraction in soil (Keiblinger et al. 2012).

**Table 1:** Examples of recent metaproteomics studies

Objective	Type of ecosystems	Type of proteomics approach	Highlights	Reference
Community structure	Contaminated environments	1D-GE or 2D-GE + LC-MS/MS	Analysis of the active community	Bruneel et al. (2011), Halter et al. (2011)
	AMD biofilm	Shotgun LC-MS/MS	Deciphering the differences between each ecotypes	Denef et al. (2010), Denef and Banfield (2012)
Functioning of ecosystems	Marine ecosystems (Ocean)	Shotgun LC-MS/MS	Effect of nutrient gradient on the community and expressed proteins	Morris et al. (2010), Sowell et al. (2011), Williams et al. (2013)
	Freshwater (Lake)	1D-GE + LC-MS/MS	Role of bacteria in carbon sulphur and nitrogen cycles	Ng et al. (2010), Lauro et al. (2011)
	Sediment impacted by AMDs	1D-GE + LC-MS/MS	Role of microorganisms in remediation, metaproteogenomics	Bertin et al. (2011), Halter et al. (2012)
	Forest soil	1D-GE + LC-MS/MS	Role of Fungi on leaf litter decomposition	Schneider et al. (2012)
	Crop Rhizospheric soil	2D-GE + LC-MS/MS	Interaction between plants and microorganisms	Wang et al. (2011)
	Semiarid solid	1D-GE + LC-MS/MS	Importance of the carbon and nitrogen fixation, nitrification, and photosynthesis	Bastida et al. (2014)
	Human microbiome	Shotgun LC-MS/MS	Markers of healthy and diseased state	Erickson et al. (2012), Ferrer et al. (2013)
Quantitative proteomics	Biofilms	Protein-SIP	Spatial and temporal variation in biofilm	Pan et al. (2011), Mueller et al. (2011), Belnap et al. (2011), Justice et al. (2014)
	Methanogenic consortium	Protein-SIP	Carbon flow	Morris et al. (2012)

##### Relative label-based quantification approaches

The 2D-GE approach is a standard proteomic approach to study relative differences in protein expression patterns when one organism is incubated in two different conditions, and therefore its adaptation capacities (Fig. 1). Because Coomassie-based staining and fluorescent labeling is correlated to the amount of proteins, it is possible to compare the protein amounts in extracts obtained in two different culture conditions by image analyses. To optimize such a comparison, the 2D Fluorescence Difference Gel Electrophoresis (DIGE) technology was developed 15 years ago (Unlü et al. 1997; Minden et al. 2009). This approach is based on the labeling of different protein extracts with different cyanines. Up to three protein samples are labeled with fluorescent dyes (for example Cy3, Cy5, Cy2) and mixed together prior to two-dimensional electrophoresis. After 2D-GE, proteins are detected by scanning the gel at excitation wavelength of each specific dye allowing the detection of each sample separately. This approach limits inter-gel variation of migration and is therefore more robust than Coomassie-stained 2D-GE. Such an approach was recently applied to community proteomics to analyze proteins specifically expressed in the presence of phenanthrene (Cébron et al. 2014).

Another way to estimate the amount of one protein in two different conditions consists in using metabolic labeling. The SILAC (stable isotopic labeling by amino acids in cell culture) approach is an example of a metabolic labeling approach in which proteins are labeled before protein extraction (Geiger et al. 2011). Incorporation of a stable isotope from any substrate labeled with ^13^C, ^15^N, or ^36^S into proteins is used in proteomic analysis of populations or communities and called protein-based stable isotope probing (protein-SIP; Seifert et al. 2012; von Bergen et al. 2013). One substrate is replaced in the cell culture by a similar substrate but with substituted stable isotopic nuclei (e.g. deuterium, ^13^C, ^15^N). Two cell populations can then be compared as they are grown in culture media that are identical except that one of them contains a “light” and the other a “heavy” form of this particular substrate. After incorporation of the substituted stable isotopic nuclei into the proteins, and protein extraction, proteins from both cell populations are combined and analyzed together by MS. Pairs of chemically identical peptides of different stable-isotope composition can be differentiated in a mass spectrometer owing to their mass difference. The quantification is based on the intensity of labeled versus non-labeled peptide signals (relative isotope abundance, RIA, labeling ratio, LR, or shape of isotope pattern, (von Bergen et al. 2013)). Metabolic labeling from an isotopically labeled substrate (Protein-SIP) was used to study differences in microcosm community structure or composition (Table 1) and allowed studying the metabolic activity of the members within an ecosystem by tracking nutrient flow (von Bergen et al. 2013). Indeed, active microorganisms present in a community incorporate labeled substrates into proteins and the identification of labeled proteins gives information on the structure of the active community and on the change in its structure. Such an approach has been used for example to study spatial and temporal variation in biofilm structures (Pan et al. 2011; Mueller et al. 2011; Belnap et al. 2011; Justice et al. 2014) or to test the carbon flow in a methanogenic consortium (Morris et al. 2012).

Other methods consist in labeling samples with tags or stable isotopes (Christoforou and Lilley 2012) and in analyzing these labeled samples using a gel-free strategy. For example, the principle of isobaric tagging approaches is to compare peptides obtained from two samples that differ only in their isotopic composition but behave identically during sample preparation, separation, and MS analysis. The technique called “isobaric tags for relative and absolute quantification” (iTRAQ) is such a multiplexed protein quantification technique (Evans et al. 2012). Peptides from protein digestions are covalently labeled on N-termini and side chain amines with tags of varying fragment masses. These labeled samples are analyzed by nanoLC-MS/MS to identify labeled peptides. The fragmentation of the attached tag generates a low molecular mass reporter ion that can be used to relatively quantify the peptides and the proteins from which they originate. Although the term “absolute” is part of its name, this approach is a relative quantification method (Evans et al. 2012). Recently, such an approach has been combined with a DIGE approach to study the protein expression in the salivary glands of *Ixodes ricinus* ticks infected by various strains of *Borrelia burgdorferi* (Cotté et al. 2014). Similar approaches are the mass-balanced, ^1^H/^2^H isotope-coded dipeptide tag (MBIT) or the quantitative proteomics with tandem mass tags approach (TMT®; Thompson et al. 2003; Yoon et al. 2014). In these techniques, several tags can be used that contain four regions, namely a mass reporter region, a cleavable linker region, a mass normalization region, and a protein reactive group. The chemical structures of all the tags are identical but each contains isotopes substituted at various positions in such a way that the mass reporter and mass normalization regions have different molecular masses in each tag. The tags can therefore be distinguished after MS/MS fragmentation. The isotope-coded affinity tag (ICAT) approach also consists in tagging proteins with isotope-coded tags (Patton et al. 2002). ICAT reagents consist of a protein-reactive group, a linker region, and a biotin tag. Two different protein samples could therefore be labeled with different tags, combined for digestion, and the labeled peptides are enriched by affinity chromatography against the tag which is part of the labeling. These enriched peptides are then analyzed by LC-MS/MS together and differential mass-tagged peptide pairs are quantified to determine the relative levels of proteins from two samples.

### Examples of proteomics approaches used in environmental microbiology

Several studies have been recently performed to characterize and quantify proteins expressed by microorganisms present within various ecosystems, such as soil, marine, and freshwater environments but also human or animal microbiome and plant-associated microorganisms (Siggins et al. 2012b). Such metaproteome datasets give important insight into microbial community structure, dynamics, and functioning (Table 1).

#### Proteomics is complementary to metagenomics to study community structures

The composition of a community is traditionally determined by the sequencing of the 16S rRNA gene. However, it is not always possible to affiliate bacteria using only 16S rRNA gene sequences (Schleifer 2009). Some microorganisms showing very similar 16S rRNA gene sequences turned out to belong to different taxa when other phylogenetic markers were used. Interestingly, the metaproteomics approach may give taxonomic information complementary to the 16S rRNA gene-based approach. Indeed, some identified peptides may be unique and specific to one species or subspecies, or semi-unique, i.e., specific to one genus. If a unique or semi-unique peptide is identified in a complex mixture, this information reveals that the species or members of this genus are present and active and thus provides valuable information on the community structure. The identification of signature peptides in orthologs has enabled the use of some proteins involved in conserved biological processes as taxonomic signatures. Such an approach was used to describe the active community at the genus level in the Carnoulès AMD (Bruneel et al. 2011), in a mildly arsenic contaminated creek (Halter et al. 2011) or in microcosms obtained from a community of phenanthrene-contaminated soil (Cébron et al. 2014).

Metaproteomic analysis coupled with deeply sampled community genomics has been a powerful tool to differentiate between close organisms and give a better view of the diversity found in a biofilm colonizing acid mine drainage (Lo et al. 2007; Simmons et al. 2008; Denef et al. 2009, 2010). Metabolomics and metaproteomic analysis of such biofilm communities were combined and led to differentiate two bacterial species from the same genus and to highlight their specific function (Wilmes et al. 2010). Altogether strain-resolved expression patterns highlight that phylogenetically close microorganisms coexist in ecosystems, sometimes belonging to the same species but with less than 1 % divergence in their nucleotide sequences of genes encoding 16S rRNA (ecotypes). At a functional level, this microdiversity leads to functional diversity, since these strains play distinct roles (Wilmes et al. 2010; Denef et al. 2010). Moreover, several studies revealed that genome recombination occurred and is crucial for the adaptation of each ecotype and that such subtle genetic variations can lead to distinct ecological functions (Lo et al. 2007; Denef et al. 2009, 2010; Denef and Banfield 2012).

#### Proteomic tools to study the functioning of communities

To study factors that may influence the community functioning, metaproteomics approaches were first performed on microcosms in laboratory-controlled conditions. Indeed, such an approach has been successfully used to analyze a laboratory-scale activated sludge system optimized for enhanced biological phosphorus removal (EBPR; Wilmes and Bond 2004). In those communities, proteins were identified belonging to an uncultured organism of the *Rhodocyclus* lineage known to accumulate polyphosphates (Wilmes and Bond 2004), but also originating from human or marine bacteria (Kuhn et al. 2011). Metaproteomics was recently applied to batch cultures in order to analyze an anaerobic microbial community degrading toluene or to study the effect of arsenic and phenanthrene on a bacterial community originating from an aged PAH and heavy-metal-contaminated soil (Jehmlich et al. 2010; Cébron et al. 2014). Similarly, the effects of temperature and exposure to trichloroethylene (TCE) on proteins expressed by the community in laboratory-scale anaerobic conditions were analyzed (Siggins et al. 2012a). These studies illustrate that metaproteomics can be used not only to describe a community in laboratory-controlled microcosms but also to study its response to perturbations. Therefore, they highlight important functions optimizing bioengineering systems. Although such studies on laboratory-scale ecosystems are crucial to understand the functioning of these communities, studying microorganisms in laboratory conditions, even in microcosm conditions, may not reflect their particular adaptation capacities in their environmental niches. Therefore, several laboratories have optimized environmental proteomics approaches to study the functioning of microorganisms within their ecosystems. In some cases, protein abundances may not necessarily correlate with protein activities, since these crucial effectors may be present but inactive. Therefore, in order to have an integrated view of an ecosystem, metaproteomics is nowadays sometimes combined with other global analyses such as metabolomics.

##### Marine and freshwater ecosystems

Metagenomics datasets have revealed the high microbial diversity of marine and freshwater communities. For example, the spatial dynamics of bacterioplankton has been evaluated along the Chesapeake Bay, the largest estuary in the United States, and the proteins identified were shown to correlate with major microbial lineages, i.e., Bacteroides and Alphaproteobacteria, present in this ecosystem (Kan et al. 2005). More recently, thousands of proteins were identified originating from bacteria, archeae, and virus in several Seas or Oceans and were compared to proteins expressed in coastal water. Shift in nutrient transport, utilization, and energy transduction along a natural nutrient concentration gradient were observed, revealing different types of organisms and expressed functions, in particular, different transporters, between both ecosystems (Sowell et al. 2009, 2011; Morris et al. 2010; Williams et al. 2013). In freshwater environments, several studies have been recently performed. For example, the effects of carbon-stimulation and fermentation-based metabolism on biogeochemical cycling or bioremediation efficiency in aquifer were investigated (Wilkins et al. 2009, 2013; Callister et al. 2010; Chourey et al. 2013; Wrighton et al. 2014). Similarly, the functioning of microbial communities was analyzed in lakes such as the Ace Lake Antartica, pointing out the role of green sulphur bacteria, actinobacteria, and cyanobacteria in sulphur, carbon, and nitrogen cycles, in such ecosystems (Ng et al. 2010; Lauro et al. 2011).

##### Terrestrial ecosystems (soil and sediments)

Terrestrial ecosystems have been the subject of several studies to better understand carbon or nitrogen cycles or effects of toxic compounds on microbial community. In the Carnoulès arsenic-rich sediments, bacterial community analyses revealed that proteins involved in the biomineralization of iron and arsenic were expressed by *Acidothiobacillus ferrooxidans* and *Thiomonas*, respectively, which supports a major role of these microorganisms in the natural attenuation of this highly contaminated environment (Bertin et al. 2011). This approach also revealed that most proteins were expressed by uncultured microorganisms belonging to a novel phylum, i.e., “*Candidatus* Fodinabacter communificans”. These bacteria may play an indirect but important role in the functioning of the ecosystem by recycling organic matter or providing other members with cofactors such as vitamins (Bertin et al. 2011). An additional study revealed that *Euglena mutabilis*, an abundant protist found in this AMD as well as in other AMDs, produces organic compounds that could serve as nutrients for bacteria (Halter et al. 2012). Finally, in the Carnoulès ecosystem, the identification of a key protein involved in the natural remediation process, the rusticyanin enzyme, was crucial to complete a reconstructed genome from an *A. ferrooxidans*-like bacterium. Thus, the use of proteomic data to refine the annotation of metagenomic data is a recent application of metaproteomics and may be defined as “metaproteogenomics” (Bertin et al. 2011). Similarly, proteogenomics was useful to highlight the key role of cytochromes variants as well as their posttranslational modifications in AMD Biofilm (Singer et al. 2010).

Protein extraction has been the limiting step to develop metaproteomics approaches on soil, due to the presence of interfering substances, the high microbial diversity present in such ecosystems and the low amount of (meta)genomic information available on microorganisms present in soils (Bastida et al. 2009; Becher et al. 2013). Recently, new protocols have been developed to identify several hundreds of proteins in crop rhizospheric soil, forest soil, or semiarid soil (Chourey et al. 2010; Wang et al. 2011; Keiblinger et al. 2012; Nicora et al. 2013; Bastida et al. 2014). Metaproteomics was also successfully used to identify the major fungi involved in leaf litter decomposition. In this study, proteins were analyzed by 1D-SDS-PAGE followed by liquid-chromatography and tandem mass-spectrometry (Schneider et al. 2012). A recent study was performed on three semiarid soils with different characteristics, testing different extraction methods (Bastida et al. 2014). This study revealed that depending on the protocols used, a taxonomic bias may be observed. Nevertheless, different community composition was observed among the three soils, and proteins involved in biogeochemical cycles of different elements were identified, revealing that the microbial communities from semiarid soils where organic carbon is limiting microbial growth, expressed proteins involved in photosynthesis, carbon and nitrogen fixation and in nitrification.

##### Eukaryotic host microbiomes (pathogens, symbionts, and commensals)

More recently, it has appeared that environmental proteomics may be crucial to identify not only proteins expressed in response to abiotic changes but also in response to biotic factors, such as those expressed by microbial hosts. The major difficulty in such studies is to distinguish microbial and host proteins, a difficulty that is reduced when the genome of both organisms is known. The second problem is to extract a sufficient amount of microbial proteins to be able to detect them. Successful studies allowed the identification of key proteins involved in virulence in several pathogens such as *Echinococcus granulosus* metacestode (Monteiro et al. 2010), *Clostridium perfringens* (Sengupta and Alam 2011), or *Anaplasma* or *Borrellia* when present in the tick vector (Ramabu et al. 2010; Cotté et al. 2014). Similarly, proteomics has been used to address the complex processes governing the interactions between symbiotic microorganisms and their host and vice versa, e.g., the adaptive response of plants interacting with mycorrhizae (Bona et al. 2011) and more recently proteins expressed by microorganisms existing in a crop rhizospheric soil (Wang et al. 2011).

Recent advances in metaproteomics also focused on animal or human microbiome. Metaproteomics was performed to study the higher termite hindgut microbiota (Burnum et al. 2011), revealing that proteins involved in carbohydrate transport and metabolism, nitrogen fixation and assimilation, energy production, and amino-acid synthesis may play a role at least as important as those involved in cellulose degradation. The number of metaproteomics studies in the field of the human intestinal tract and oral cavity recently increased due to the progresses made to generate metagenomic data (Human Microbiome Project Consortium 2012). For example, catalogues comprising several thousands of proteins were obtained by shotgun proteomics from human fecal samples or oral cavity (Verberkmoes et al. 2009; Rooijers et al. 2011; Kolmeder et al. 2012; Jagtap et al. 2012). In this field, metaproteomics will probably lead to the identification of protein markers of healthy or diseased states (Erickson et al. 2012; Juste et al. 2014), or to understand how microorganisms interact together or with their host in such ecosystems (Ferrer et al. 2013).

### New developments in community proteomics: targeted proteomics approaches applied to community studies and environmental microbiology

For the past 20 years, global proteomic approaches have enabled the identification and relative quantification of ever-increasing lists of proteins, in all kinds of complex protein mixtures from plasma to cell lysates to bacterial communities. More recently, targeted proteomics approaches have been developed as an alternative to global approaches with the aim of trying to find ways to more precisely quantify a subset of proteins, even if this implies limiting the focus on a restricted number of proteins of interest. Selected or multiple reaction monitoring mass spectrometry (SRM or MRM) appeared to be the most promising approach to achieve this goal (Picotti and Aebersold 2012) and many applications emerged, mainly in the field of biomarker verification (Gillette and Carr 2013; Percy et al. 2014). To set up a targeted LC-SRM assay, the prior definition of a short list of proteins of interest is necessary. This is the main difference between global (in which the goal is to identify the largest number of peptides/proteins) and targeted approaches in which the protein targets must be defined prior to the experiment itself. Once the targets are defined, a few peptides whose sequences are specific for the target protein and visible in MS, called proteotypic peptides, will be selected to be used as tracers to quantify the corresponding protein.

Briefly, LC-SRM experiments are mainly conducted on triple quadrupole-type instruments, using the first quadrupole to select a precursor ion, the second quadrupole to fragment this precursor ion and the third quadrupole to select a specific fragment ion. A predefined pair of precursor/fragment ions is called a transition and multiple transitions are measured for each target peptide. The appropriate selection of proteotypic peptides and their most sensitive and specific (interference-free) transitions is a crucial step to reach sensitivity and specificity required for a robust and high-performance quantitative LC-SRM assay. To facilitate this selection, public libraries (atlases) built on synthetic peptides for reference proteomes, namely yeast, *Mycobacterium tuberculosis*, and human are available (http://www.srmatlas.org; Picotti et al. 2008; Schubert et al. 2013). Besides proteotypic peptide sequences for all proteins of these reference proteomes, the atlases also include important instrument parameters enabling a faster assay development, namely relative peptide retention times, optimal collision energies, or interface voltages. These instrument parameters need to be optimized in any case and this optimization is usually done on crude synthetic, ideally heavy labeled, peptides that need to be synthetized when information in the atlases is missing or incomplete. In the case of bacterial communities, no atlas is available and the possibility to get access to low-cost crude synthetic peptides is therefore very important.

A key factor for reliable quantification is the use of appropriate standards. Indeed, attempting to reach absolute quantification of peptides is possible only through simultaneous analysis by LC-SRM of endogenous peptides and labeled peptides added in known amounts. Highly purified and precisely quantified heavy labeled synthetic standards exist in several alternative forms: purified and quantified synthetic peptides (AQUA peptides, Gerber et al. 2003), concatemers of peptides (QconCAT, Beynon et al. 2005) and standard proteins biochemically identical to natural proteins (PSAQ, Protein Standard Absolute Quantification, Brun et al. 2007). For precise absolute quantification, endogenous peptides of interest are quantified by calculating heavy/light peptide ratios thanks to spiked heavy-labeled synthetic peptides into the samples (Lange et al. 2008; Gallien et al. 2011; Fig. 2). Isotope dilution is used for the quantification of small molecules such as metabolites, xenobiotics, hormones, or pesticides with high precision (CV <5 %) for more than 30 years. Today, the main obstacles for the application of SRM to quantify peptides and proteins start to be overcome: (1) high sensitivity has been reached thanks to significant instrument improvements, (2) large dynamic ranges are accessible thanks to the implementation of reproducible fractionation steps prior to LC-SRM analysis, (3) multiplexing capacity has been increased thanks to significant advances in electronics, data acquisition, and software developed on triple quadrupole and high performance liquid chromatography instruments but also thanks to the implementation of retention time reference peptides workflows (Escher et al. 2012). Alternatively to LC-SRM methods, the high resolution of different instrument geometries (Quadrupole-Orbitrap or Quadrupole-Time Of Flight instruments) is explored to develop targeted methods for precise quantification. Rather than following isolated fragments from the precursors of interest as SRM does, these methods rely on the acquisition of full scan high resolution MS/MS fragmentation spectra of the peptides of interest (Gallien et al. 2012).

So far, most targeted proteomics applications dealt with biomarker studies and clinical proteomics (Hüttenhain et al. 2012; Gillette and Carr 2013; Percy et al. 2014). A very recent study demonstrated the application of LC-SRM assays to verify markers of Crohn’s disease, discovered by 2D-DIGE experiments, in unfractionated gut microbiota (Juste et al. 2014). Besides, a study has demonstrated the ability to absolutely quantify proteins in complex environmental samples and mixed microbial communities (Werner et al. 2009). Such an approach will be probably extensively used in the next future to follow the activity of specific targeted bacteria within communities.

## Conclusion

Nowadays metaproteomics is usually combined with other high-throughput “omics” methods, such as metatransciptomics, metametabolomics as well as more traditional methods of genetics, molecular biology and/or biochemistry. All generated data are combined in order to decipher ecosystem functioning and give an integrated view of biological objects in any environment, their roles, and relationships. Such global studies of microbial communities will be of great interest to investigate complex consortia and address the role of uncultivated microorganisms in microbial ecosystems. In the future, these approaches will be further improved in order to access proteins expressed by individual cells within a community, which will give important insight into the community structure and its functioning. The recent developments in quantitative and targeted community proteomics will open further possibilities to decipher which factors modify their dynamics. Therefore, proteomics should lead not only to a better understanding of ecosystems themselves, but also to the identification of new functions that can be exploited in biotechnological applications. This could lead to an optimal use of the properties of microorganisms and to a better understanding of how microorganisms colonize new ecological niches.
